# Supplementary material for: Anticancer effect of zanubrutinib in HER2-positive breast cancer cell lines
Source: Invest New Drugs. 2023 Mar 13;41(2):210–9. doi: 10.1007/s10637-023-01346-7 (PMC10140101; doi:10.1007/s10637-023-01346-7)
Supplement: Supplementary file 1 — Supplementary Material 1 [file 10637_2023_1346_MOESM1_ESM.docx]

**SUPPLEMENTARY INFORMATION**

**Anticancer effect of zanubrutinib in HER2-positive breast cancer cell lines**

Hana Dostálová^1^, Radek Jorda^1^, Eva Řezníčková^1^, Vladimír Kryštof^1,2^

**^1^** Department of Experimental Biology, Faculty of Science, Palacký University Olomouc, Šlechtitelů 27, 78371 Olomouc, Czech Republic

**^2^** Institute of Molecular and Translational Medicine, Faculty of Medicine and Dentistry, Palacký University Olomouc, Hněvotínská 5, 77900 Olomouc, Czech Republic

Corresponding author: Vladimír Kryštof, vladimir.krystof@upol.cz

Supplementary table 1: Percent inhibition of ERBB kinases by BTK inhibitors (1 µM)

Supplementary figure 1: HER2 and BTK status verification of chosen breast cancer cell lines either via immunoblotting (A) or FISH (B)

Supplementary figure 2: Effects of BTK inhibitors on ERBB downstream target in HER2 negative breast cancer cell line MCF7

Supplementary figure 3: BTK inhibitors show no significant impact on cell cycle progression in HER2 negative cell line MCF7

**Supplementary table 1**. Percent inhibition of ERBB kinases by BTK inhibitors (1 µM). Data extracted from published literature

| **Inhibitor [ref.]** | **EGFR** | **HER2** | **HER4** |
| --- | --- | --- | --- |
| Ibrutinib [1] | 89 | 91 | 94 |
| Acalabrutinib [1] | -1 | 36 | 93 |
| Zanubrutinib [2] | 86 | 40 | 96 |
| Tirabrutinib [1] | -2 | 7 | 28 |
| Evobrutinib [1] | 1 | 1 | 57 |


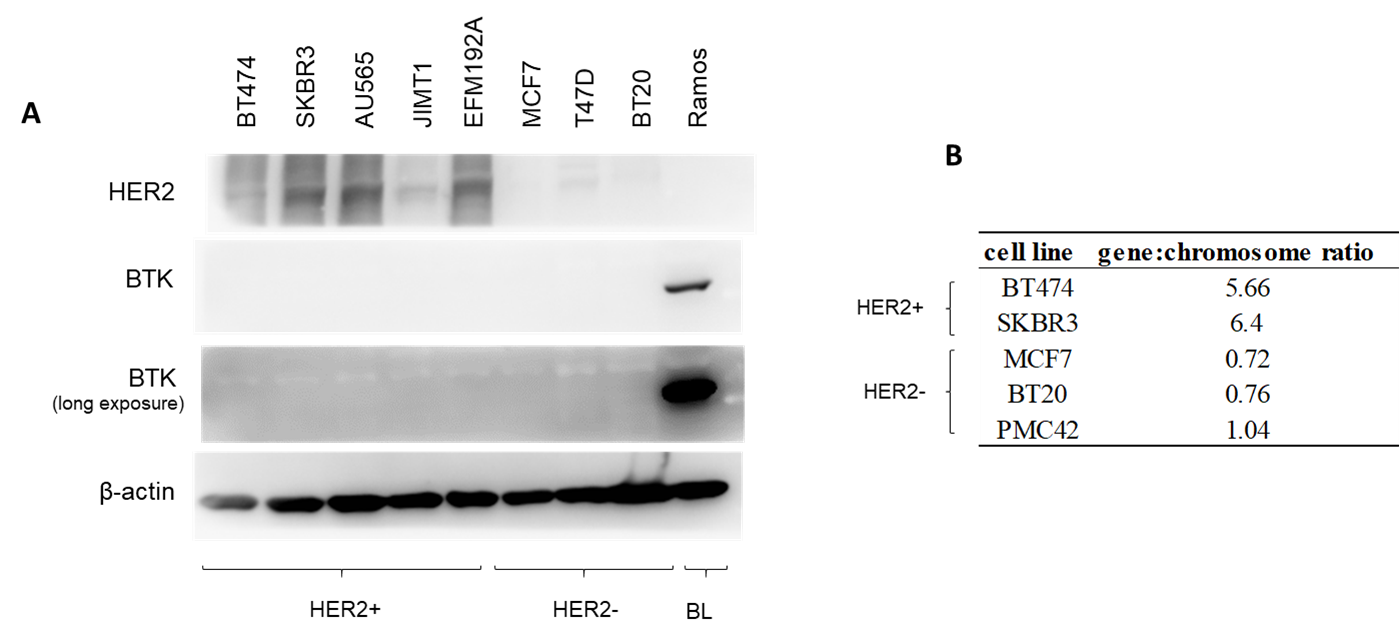


**Supplementary fig. 1** HER2 and BTK status verification of chosen breast cancer cell lines either via immunoblotting (A) or FISH (B). β-Actin served as control of equal loading. The representative results from at least two biological replicates are shown. BL –Burkitt’s lymphoma.

FISH was performed according to the manufacturer’s instructions with the PathVysion HER-2 DNA Probe Kit (Vysis, Downers Grove, IL). The data were obtained and analysed as described in previous publications [3]. Briefly, the number of gene/centromere signals was counted in 100 non-overlapping nuclei in each cell line. Samples were classified as amplified if the *HER2*/CEP17 ratio was ≥2.0 and/or the mean *HER2* copy number was ≥6, equivocal if the *HER2*/CEP17 ratio was <2.0 and the mean *HER2* copy number was between 4 and 6, and negative if the *HER2*/CEP17 ratio was <2.0 and the mean *HER2* copy number was <4.


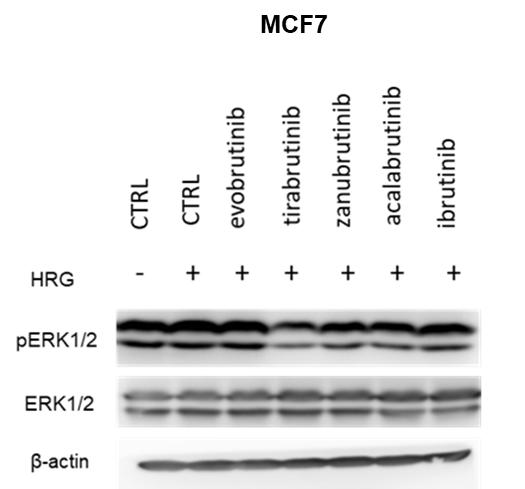


**Supplementary fig. 2** The effects of BTK inhibitors on ERBB downstream target in HER2 negative breast cancer cell line MCF7. Compounds were used in 10 µM concentration for 16 hour treatment. Cells were stimulated by heregulin (HRG, 0.1 µg/ml) 30 min prior to harvesting. β-Actin served as control of equal loading. The representative results from at least two biological replicates are shown.


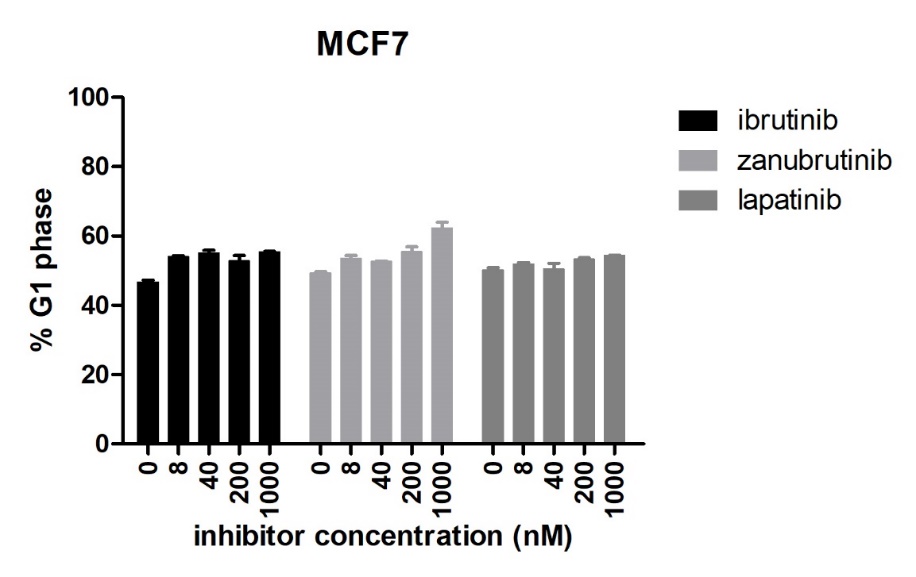


**Supplementary fig 3** BTK inhibitors show no significant impact on cell cycle progression in HER2 negative cell line MCF7. Cells were treated with indicated concentrations of selected compounds for 24 hours. Error bars represent standard deviations, data are averages of biological triplicates

**References**

1. Crawford JJ, Johnson AR, Misner DL, et al (2018) Discovery of GDC-0853: A Potent, Selective, and Noncovalent Bruton’s Tyrosine Kinase Inhibitor in Early Clinical Development. J Med Chem 61:2227–2245. https://doi.org/10.1021/acs.jmedchem.7b01712

2. Guo Y, Liu Y, Hu N, et al (2019) Discovery of Zanubrutinib (BGB-3111), a Novel, Potent, and Selective Covalent Inhibitor of Bruton’s Tyrosine Kinase. J Med Chem 62:7923–7940. https://doi.org/10.1021/acs.jmedchem.9b00687

3. Koudelakova V, Trojanec R, Vrbkova J, et al (2016) Frequency of chromosome 17 polysomy in relation to CEP17 copy number in a large breast cancer cohort. Genes, Chromosom Cancer 55:409–417. https://doi.org/10.1002/gcc.22337
